# Examining the role of funders in ensuring value and reducing waste in research: An organizational case-study of the Patient-Centered Outcomes Research Institute

**DOI:** 10.12688/f1000research.18471.2

**Published:** 2019-06-11

**Authors:** Evelyn P. Whitlock, Joe V. Selby, Kelly M. Dunham, Alicia Fernandez, Laura P. Forsythe, Grayson Norquist

**Affiliations:** 1 Patient-Centered Outcomes Research Institute, Washington, DC, 20036, USA; 2 San Francisco School of Medicine, University of California San Francisco Medical Center, San Francisco, CA, 94143, USA; 3 Department of Psychiatry and Behavioral Sciences, Emory University School of Medicine, Atlanta, GA, 30329, USA

**Keywords:** Biomedical Research/standards, Research Design/standards, Biomedical Research/economics, Biomedical Research/methods

## Abstract

International experts have recommended actions that funders can take to improve the value of research investments. They state that self-assessment and public sharing are the basis for accountability and improvement. We examined our policies and practice to determine the extent to which the Patient-Centered Outcomes Research Institute’s (PCORI) policies and practices as a research funder align with international best practice recommendations. A self-audit of current policies and practice against 17 recommendations and 35 sub-recommendations representing five major stages of research production, based on adapted methods used for self-assessment by another funder, was performed.  Fit of existing PCORI policies and practices with 35 sub-recommendations, qualitative assessment of adequacy (area of strength; area of partial strength; area of growth; not applicable) for 17 recommendations for five stages of research production was assessed. Of the 17 recommendations, 15 were applicable to PCORI’s research mission and focus.  PCORI has policies and practices in place for all elements of six recommendations (“area of strength”) and policies that address each element but with some still in active development for three (“area of partial strength”). PCORI is partially addressing six of the 15 relevant recommendations (“area of growth”). Areas for growth include making study protocols publicly available, improving policies on data sharing, and enhancing collaboration with other funders to reduce redundant funding. A voluntary consortium of international funders is underway to encourage further progress, including additional self-assessment and public sharing for accountability. These findings indicate PCORI has undertaken efforts to align its funding practices with international recommendations to ensure the value of public dollars invested in research.  Further efforts will likely require additional coordination and collaboration between funders and stakeholders.

## Introduction

In 2014, in response to concerns about avoidable waste in research prioritization, conduct, and reporting ^1^, *The Lancet* published a series of articles which identified specific recommendations for the biomedical research community to ensure value and minimize inefficiency in research ^2– 6^. Research funders were a major target for these recommendations, along with regulators, journals, academic institutions and researchers themselves. Prompted by these and related activities, the biomedical research community around the world has begun considering best practices to ensure value in publicly-funded research. As key contributors ^7^, research funders are encouraged to audit and update their own policies and practice, even as external assessments of funders are also undertaken ^8, 9^.

 In light of these trends, the Patient-Centered Outcomes Research Institute (PCORI), undertook an organizational case study of its policies and practices. PCORI was created in 2010 to address research needs of a range of healthcare stakeholders through clinical comparative effectiveness research, and ranks among the top 10 US non-commercial funders of health research (see Healthresearchfunders.org). Our goals were to examine and report how closely PCORI adheres to best practice recommendations for research funders (i.e., to foster transparency), to highlight areas of needed development for PCORI (to foster public accountability), and to consider how other research funders in the US and elsewhere can examine, report, and adopt best practices for supporting value in research (to foster enterprise-wide efficiency).

## Methods

To maximize comparability, we adapted another funder’s self-assessment methods (M. Westmore, personal communication, June 15, 2016; See Adding Value in Research from the National Institute for Health Research). PCORI staff (KD, LF, EW) examined PCORI’s existing policies and initiatives against 17 recommendations for funding agencies from the *Lancet* series ^2– 6^; after initial assessment, we consulted with additional PCORI staff members to confirm accurate interpretation of policies and processes (see PCORI site). Many of the 17 *Lancet* recommendations include multiple components. To accurately assess and transparently communicate our performance across all intended components of these recommendations, we subdivided some recommendations to capture each dimension within them separately, for a total of 35 sub-recommendations. ( Table 1). Four authors (KD, LF, EW, GN) independently categorized fidelity to the 17 recommendations as: 1) “area of strength” –PCORI’s practices reasonably address all sub-recommendations; 2) “area of partial strength” –PCORI’s practices reasonably or partially address all sub-recommendations; 3) “area of growth” –PCORI’s practices do not address all sub-recommendations, either reasonably or partially; or 4) not applicable. We resolved discrepancies through discussion and final ratings reflect consensus.

## Results

Table 1 represents a detailed summary (through November 2018) of PCORI’s policies and practices related to ensuring value in research. Across the 17 recommendations (35 sub-recommendations), two recommendations were not applicable (1, 8), and one recommendation primarily applies to non-funders (both 9a, 9b). For the 15 relevant recommendations, PCORI at least partially addresses most of the relevant sub-recommendations (28/33). Our consensus process categorized PCORI’s existing policies and practices as “areas of strength” for 6/15 applicable recommendations, “partial strength” for 3/15. PCORI’s authorizing legislation, although preceding the Lancet recommendations by several years, mandated a number of these (indicated in bold in the table). 

**Table 1.** Assessment of PCORI’s policies and practices related to ensuring value in research.

LANCET SERIES RECOMMENDATIONS	RELATED PROCESSES OR INITIATIVES AT PATIENT-CENTERED OUTCOMES RESEARCH INSTITUTE (PCORI)	SELF-ASSESSMENT
QUESTIONS ARE RELEVANT TO USERS OF RESEARCH		
1. More investigations into research should be done to identify factors associated with successful replication of basic research and translation to application in health care, and how to achieve the most productive ratio of basic to applied research	• Not applicable - **PCORI is authorized to fund comparative** **clinical effectiveness research with direct application to** **health care decision making**.	Not applicable
2. Research funders should make information available about how decisions are made about what research to support (2a) and fund investigations into the effects of initiatives to engage potential users of research in research prioritization (2b)	** • PCORI’s multi-stakeholder Board of Governors meetings are** **conducted in an open forum, allowing members of the public** **to listen and provide comment on the proceedings.** (2a) • PCORI incorporates potential users of research throughout the decision-making process from identification of research priorities to topic refinement, in review of applications for research funding. PCORI publishes criteria and processes guiding the topic pathway which determines focused research funding opportunities. (2a) • PCORI’s Engagement Awards provide funding for patient and stakeholder groups for prioritization of research topics. (2b) • PCORI has funded studies focused on improving the methods for research prioritization. (2b)	Area of strength
3. Research funders and regulators should demand that proposals for additional primary research are justified by systematic reviews (3a), showing what is already known (3b), and increase funding for the syntheses of existing evidence (3c)	• PCORI’s Methodology Standards require that any proposed study be justified by evidence gaps identified through gap analysis or systematic review. (3a) • The first of the PCORI application Merit Review Criteria requires that applications demonstrate the potential for the study to fill critical gaps in evidence. (3a; 3b) • **PCORI’s legislation specifies its use of evidence synthesis** **to increase quality and relevance of information;** programs for funding evidence syntheses have been expanding since 2016 including systematic reviews and updates, individual patient data meta-analysis and other evidence synthesis approaches. (3c) • PCORI has funded studies on improving methods for systematic reviews. (3c)	Area of partial strength
4. Research funders and research regulators should strengthen and develop sources of information about in progress research (4a), ensure that this information is used by researchers (4b), insist on publication of protocols at study inception (4c), and encourage collaboration to reduce waste (4d)	• Abstracts and project statuses for all research awards are available on PCORI’s website with links to project registration in clinicaltrials.gov, PROSPERO, and Registry of Patient Registries (RoPR). (4a) • PCORI launched several topic-based, multi-stakeholder networks to increase cross-learning, information sharing, collaboration, and uptake of findings. (4d) • PCORI consults with other US funders when considering new research topics and initiatives to prevent duplication and identify areas for collaboration or co-funding. (4d)	Area of growth
APPROPRIATE RESEARCH DESIGN, CONDUCT, AND ANALYSIS ARE EMPLOYED		
5. Make publicly available the full protocols (5a), analysis plans or sequence of analytical choices (5b), and raw data (5c) for all designed and undertaken biomedical research	• **PCORI’s authorizing legislation requires that research** **protocols, methods of research and analysis, and other** **information be made publicly available concurrent with the** **release of research findings**. (5a; 5b) • PCORI’s policy on Replication and Reproducibility of Research and Data Sharing requires awardees to submit the final protocol to PCORI, requires applicants/awardees to submit a data- sharing plan, and permits PCORI to request data sharing and to share protocols upon request. (5a; 5b; 5c)	Area of growth
6. Maximize the effect to bias ratio in research through: defensible design and conduct standards (6a), a well-trained methodological research workforce (6b), continuing professional development (6c), and involvement of non-conflicted stakeholders (6d)	• **PCORI established a set of Methodology Standards for** **relevant research designs and requires that all PCORI-** **funded research adhere to relevant PCORI Methodology** **Standards**. (6a) • PCORI offers training opportunities to develop the research workforce and support researchers in understanding and applying the Methodology Standards, including continuing medical education (CME). (6b; 6c) • **PCORI’s requires that any conflicts of interest be disclosed** **for advisory panel members, individuals involved in the** **peer-review process, Board and Methodology Committee,** **and for executive staff of the Institute**. PCORI’s Policy on Conflict of Interest (COI), Confidentiality, and Non-Disclosure ensures that application merit reviewers provide objective evaluations of applications for funding. (6d) • Individual patient data meta-analyses are set up to involve third party researchers with strict COI consideration consistent with guidance from the Institute of Medicine and the World Health Organization. (6d)	Area of strength
7. Reward (with funding and academic or other recognition) reproducibility practices and reproducible research and enable an efficient culture for replication of research	Not yet developed	Area of growth
RESEARCH REGULATION AND MANAGEMENT IS EFFICIENT		
8. People regulating research should use their influence to reduce other causes of waste and inefficiency in research	• Not applicable –PCORI does not regulate research.	Not applicable
9. Regulators and policy makers should work with researchers, patients, and health professionals to streamline and harmonize the laws, regulations, guidelines, and processes that govern whether and how research can be done (9a), and ensure that these factors are proportionate to the plausible risks associated with the research (9b)	• PCORI follows practices recommended by Office for Human Research Protections, National Institutes of Health, and seeks counsel to harmonize human subject protections. (9a; 9b) • Accelerating Patient-Centered Outcomes Research and Methodological Research is one of PCORI’s five national priorities for research. PCORI’s Methods Program is funding research on novel approaches to improving research efficiency in informed consent (9a; 9b) • PCORnet, the national patient-centered clinical research network launched by PCORI, has done extensive work related to multi-institutional contracting, IRB oversight, data sharing and data linkage. (9a, 9b)	Area of partial strength
10. Researchers and research managers should increase the efficiency of recruitment and retention of participants, data monitoring, and data sharing in research through the use of research designs known to reduce inefficiencies (10a), and do additional research to learn how efficiency can be increased (10b)	• **PCORI’s multi-stakeholder Advisory Panel on Clinical** **Trials advises PCORI on the selection, research design,** **implementation, and technical issues of clinical trials** **for patient-centered outcomes research.** Recruitment subcommittee advises on strategies for appropriate patient recruitment, accrual and retention of participants in clinical trials. (10a) • PCORI launched the National Patient-Centered Clinical Research Network (PCORnet), a collaboration involving 33 individual partner networks, intended to conduct clinical research incorporating patient health information more efficiently and at lower cost than is currently possible; intended efficiencies in recruitment, retention, data monitoring and data sharing still being explored. (10b) • PCORI is funding research on novel approaches to improving research efficiency in recruitment, appropriate use of observational data for valid causal inference, and methods to allow for real-world clinical research. (10b)	Area of partial strength
11. Everyone, particularly individuals responsible for health-care systems, can help to improve the efficiency of clinical research by promoting integration of research in everyday clinical practice	• PCORI promotes integration of research in everyday clinical practice through: • Demonstration: 139 healthcare sites are embedded within PCORnet sites • Funding: PCORI conducts many pragmatic studies in real- world settings • Training: PCORI and the Agency for Healthcare Research and Quality are co-funding workforce training within learning health systems. • Dissemination: PCORI works with stakeholders, including healthcare systems, to promote dissemination and implementation of key research findings and provides competitive funding opportunities for these purposes.	Area of strength
ALL RESEARCH IS REPORTED AND DATA ARE ACCESSIBLE		
12. Institutions and funders should adopt performance metrics that recognize full dissemination of research (12a) and reuse of original datasets by external researchers (12b)	• **PCORI-funded research findings are required to convey** **full results including considerations specific to certain** **subpopulations and study limitations**. (12a) • PCORI’s Board of Governors monitors high-level performance metrics on dissemination of research findings. (12a) • PCORI has developed initial Data Management and Data Sharing policies but not performance measures. (12b)	Area of growth
13. Investigators, funders, sponsors, regulators, research ethics committees, and journals should systematically develop and adopt standards for the content of study protocols (13a) and full study reports (13b), and for data sharing practices (13c)	• Awardees submit final research reports using a standard template designed to increase the quality and transparency of reporting. Awardees are required to consider the PCORI Methodology Standards for data integrity, rigorous analyses, and reporting and to follow international checklists for reporting and assessing quality (e.g. CONSORT, STROBE, PRISMA). (13b) • PCORI’s Policy on Data Management and Data Sharing was approved by the PCORI Board of Governors on September 7, 2018. The Policy was informed, in part, by a pilot project that brought together PCORI awardees with data repository organizations. (13c)	Area of growth
14. Funders, sponsors, regulators, research ethics committees, journals, and legislators should endorse and enforce study registration policies (14a), wide availability of full study information (14b), and sharing of participant-level data for all health research (14c)	• PCORI requires awardees to register awards in clinicaltrials.gov, PROSPERO, and Registry of Patient Registries (RoPR) (14a) • PCORI’s Public Access to Journal Articles policy provides funds for all projects to cover open access fees and requires all publications to be deposited in PubMed Central (PMC) (14b)	Area of growth
RESEARCH REPORTS ARE COMPLETE, UNBIASED, AND USABLE		
15. Funders and research institutions must shift research regulations and rewards to align with better and more complete reporting	• **PCORI findings are required to undergo a Peer Review** **process assessing scientific quality and level of adherence** **to PCORI methodology standards prior to publication**. • Receipt of funds dependent on completion of contract milestones, including registration of study on clinicaltrials.gov and completion of final peer-reviewed report to be posted on PCORI website.	Area of strength
16. Research funders should take responsibility for reporting infrastructure that supports good reporting and archiving	• **Lay and Clinician Abstracts that are comprehensible, useful,** **fully convey findings, discuss considerations specific to** **subpopulations, and address limitations as well as research** **needs are required by law to be available on PCORI website** **within 90 days of study completion**. • The PCORI website provides infrastructure for transparent reporting. Audio files, Spanish translations, and other translation products aid PCORI in the dissemination of research findings.	Area of strength
17. Funders, institutions, and publishers should improve for authors and reviewers the capability and capacity for high-quality and complete reporting	• PCORI’s Peer Review process assesses scientific quality and level of adherence to PCORI methodology standards prior to publication of research findings. Reviewers provide feedback to study investigators to ensure complete reporting.	Area of strength

Recommendations sourced from Lancet series on ensuring value and minimizing waste in research ^2– 6^ Bolded text indicates process or policy mandated by PCORI’s authorizing legislation

## Discussion

Our consensus process categorized PCORI’s existing policies and practices as meeting criteria for “areas of strength” or “partial strength” for many of the recommendations, and we also identified clear areas for growth. Examples of strengths include PCORI’s requirements that funded research adhere to methodology standards to minimize bias and that all study results are posted on the PCORI website to enhance public access to findings. On the other hand, PCORI has not yet fully developed its policies and practices related to rewarding research replication and reproducibility (Recommendation 7). Further development of performance metrics, standardized approaches to all study-related reporting, and enforcement of key policies (Recommendations 12, 13, 14) offer other areas ripe for growth, particularly if undertaken in coordination with others across the research enterprise. PCORI like many funders, is still actively developing its practices related to publicly sharing information, including raw data, as early as possible from funded research (Recommendations 4, 5). For example, making research protocols publicly available (Recommendation 5a) is required by PCORI’s authorizing legislation, but timing and format were not specified, and our current practices may not be ideal. PCORI now requires funded investigators to submit a study protocol and record its details in an appropriate registry but does not yet specify a standard protocol format nor require protocol publication before study completion. To our knowledge, just one funder (NIHR) clearly publishes study protocols at the time of award ^10^. Nonetheless, making study protocols available at study inception can benefit the public by providing a detailed record of the planned study, which may help avoid unwitting duplication of research underway and support detection of important study deviations and post-hoc changes. 

There is also opportunity for improvement through further development of policies and practices related to research data sharing and re-use. While funders can require awardees to share data from funded research and trial participants are supportive of such sharing ^11^, many researchers remain concerned about the impact on their work ^12^. PCORI’s policy on data sharing ^13^ was informed by a public comment process as well as pilot work assessing time and effort required for investigators to prepare their data for sharing and on identifying appropriate repository models. Accelerating the practice of responsible data sharing necessitates broad coordination between journals, academic institutions, and data-repository organizations, alongside consistent requirements and support from funders. PCORI plans to monitor progress in these areas and conduct an updated self-assessment in two years.

Efforts to reduce waste and increase value in research are in alignment with trials transparency ^14^, research integrity ^15^, administrative efficiency ^16^, and other similar initiatives. PCORI and other health research funders are in consortium to encourage further development and voluntary adherence to international best practice recommendations for research funders, ( 17; see Ensuring Value in Research (EVIR) website). The Ensuring Value in Research Funders’ Forum is exploring other initiatives, such as evaluating and sharing best practices for similar challenges that funders face, and considering what avenues exist to enhance efficiency and value in the full research agenda across funders. Beyond the consortium, greater transparency and coherence between funders and key players producing health research---including journals, research institutions, sponsors, and regulators---remains vital for tangible progress in our shared efforts ^7, 18^.

Limitations: Our methods are limited by self-assessment, but findings are consistent with audit results for PCORI from external assessors ^10^. In addition, the availability of policies or current practices represent only the first step, with actual performance measurement needed. Finally, while the Lancet series highlights areas for improvement for funders and others across the research enterprise, the impact of implementing and adhering to these recommendations on research value has yet to be demonstrated. 

## Data availability

### Underlying data

All data underlying the results are available as part of the article and no additional source data are required.
