# Supplementary material for: Examining the role of funders in ensuring value and reducing waste in research: An organizational case-study of the Patient-Centered Outcomes Research Institute
Source: F1000Res. 2019 Jun 11;8:288. Originally published 2019 Mar 14. [Version 2] doi: 10.12688/f1000research.18471.2 (PMC6518438; doi:10.12688/f1000research.18471.2)
Supplement: Peer review history

# Peer review: Reviewer response for version 2

**DOI:** 10.5256/f1000research.21339.r49744

**Published:** 2019-06-11

**Authors:** Mona Nasser

**Affiliations:** 1 Peninsula Dental School, Faculty of Medicine and Dentistry, University of Plymouth, Plymouth, UK

No further comments

I have read this submission. I believe that I have an appropriate level of expertise to confirm that it is of an acceptable scientific standard.

# Peer review: Reviewer response for version 1

**DOI:** 10.5256/f1000research.20209.r45788

**Published:** 2019-05-14

**Authors:** Mona Nasser

**Affiliations:** 1 Peninsula Dental School, Faculty of Medicine and Dentistry, University of Plymouth, Plymouth, UK

This is a very important project and the authors have put a lot of effort to ensure a structured approach to self-evaluation of the organisation.

 It would be helpful to have more details on the methods:

- Did all four authors go through all questions and sections, or was it divided between them with at least two people independently having seen each section?
- Did individuals respond to the question based on their experience or knowledge, or did it additionally involve any review of policies and documents internally by them?
- I was not sure what you meant by “reasonably” – did you mean that PCORI has generally met the recommendation, even if there is room for improvement available (unlike “area of growth” which is a gap and needs improvement).
- The table provides a very good summary but in some cases like area 4 and 12, it isn’t clear where the areas of growth are, so would be helpful to explain it (which is the part I mentioned is missing from the data to be able to reproduce).

I have read this submission. I believe that I have an appropriate level of expertise to confirm that it is of an acceptable scientific standard.

# Response

**Published:** 2019-05-24

**Authors:** Kelly Dunham

**Affiliations:** PCORI, USA

We greatly appreciate the reviewer's questions and suggestions. We made several revisions to the article in response to the comments.

 At least two PCORI staff members reviewed each recommendation. Policies and documents were reviewed, where applicable, in addition to knowledge-based documentation. Our assessments were confirmed by other staff who were not involved as co-authors, particularly for policies and procedures not centrally documented. 

 We appreciate the reviewer's question about the meaning of "reasonably." We employed the term "reasonably" to indicate two things. First, that PCORI has generally met the recommendation. Second, that any assessment made relies on a qualitative judgement, but one that we sought to measure against a standard of reasonableness, i.e., "reasonable people would come to the same conclusion looking at the same information." Thank you for this question, which allows us to provide this further clarification. 

 We appreciate the request to be explicit, and made an effort to do so with examples in the text for areas like making the protocol publicly available (Recommendation 5a) and data sharing (Recommendations 12-14). We are limited in our ability to be completely explicit across all areas of growth for several reasons: 1) in some areas, such as ensuring the complete availability and sharing of primary study data, practices for funders and other actors are less established and more developmental than in other domains. We don't have clear standards against which to assign specific areas of growth; and 2) some areas of growth require strategic decisions and direction by governing bodies, once identified. For these areas,  we cannot articulate next steps without a more deliberative process which this audit and resultant activities should help stimulate. 

 Thanks again for the thoughtful critique; your comments have greatly improved the article.

# Peer review: Reviewer response for version 1

**DOI:** 10.5256/f1000research.20209.r47387

**Published:** 2019-04-25

**Authors:** Hans Lund

**Affiliations:** 1 Centre for Evidence-Based Practice, Western Norway University of Applied Sciences (HVL), Bergen, Norway

The study is a report of a self-audit done by one of the top 10 non-commercial funders in USA. The aim was to evaluate to what degree the funding agency follow international recommendations to improve the value of research investments.

 This self-audit is very important for a general audience as:

1. Researchers can understand the context and environment of funding and the reason for the requirements related to application for funding
2. Other funding agencies can see how to change their policies in order to improve the value of research investments
3. Readers will understand the challenges related to improve the value of research investments

The report should include a date for when to expect an update of the self-audit. An update - for example 2 to 3 years from now - would show the improvements and identify the biggest challenges related to improve the value of research investments.

 The method is only partly described as the reader is unable to see from where the 17 recommendations and 35 subrecommendations originates. None of the link leads the reader directly to the source. In addition, as the authors use another funder´s self-assessment method, the possible alterations or adjustments made in the present self-audit should be mentioned. If no alterations were done, this should also be mentioned. Using the same assessment method makes is possible to compare, and this could have been mentioned in the Discussion.

 There is an * and a † in Table 1, I can´t find what these refers to.

 In conclusion: this is a very important and useful report of a self-audit (see above), and with the minor adjustments mentioned is should be published the sooner the better.

I have read this submission. I believe that I have an appropriate level of expertise to confirm that it is of an acceptable scientific standard.

# Response

**Published:** 2019-05-24

**Authors:** Kelly Dunham

**Affiliations:** PCORI, USA

We greatly appreciate the reviewer's comments and suggestions. 

While we routinely monitor PCORI practice, we plan to conduct a second self-assessment in two years. We added this information to the discussion section.

We appreciate the reviewer's suggestion regarding the methods section. In the revised article, we explicitly linked to the 17 recommendations from the *Lancet *series. We  made clearer in the text that we broke these into a total of 35 sub-recommendations, since the original 17 sometimes included multiple items against which it would have been difficult to assess and transparently communicate our current activities. In other words, it would have been easy to claim credit for doing one part of a recommendation when we were not addressing another aspect of the recommendation at all. 

The other funder's self-assessment was not published, so it cannot be linked to directly. We added a reference to personal communication in the revised article. We did not have a copy of their written methods so cannot make the clear comparison that is requested, but have made our approach more transparent to avoid confusion.

Thank you for pointing out the miscellaneous symbols in the table. These were holdovers from a previous version of the table and have been removed. 

Thank you for the thoughtful critique. Your comments and questions have helped us to clarify the methods used and greatly improved the article.
